# Breastfeeding and COVID-19 vaccination: position statement of the Italian scientific societies

**DOI:** 10.1186/s13052-021-00998-6

**Published:** 2021-02-27

**Authors:** Riccardo Davanzo, Massimo Agosti, Irene Cetin, Antonio Chiantera, Giovanni Corsello, Luca A. Ramenghi, Annamaria Staiano, Marcello Tavio, Alberto Villani, Elsa Viora, Fabio Mosca

**Affiliations:** 1 Institute for Maternal and Child Health - IRCCS Burlo Garofolo, Via dell’Istria 65/1, 34100 Trieste, Italy; 2 Woman and Child Department, Ospedale Del Ponte, Varese, Italy; 3 Department of Woman, Mother and Neonate, Vittore Buzzi Hospital, University of Milan, Milan, Italy; 4 SIGO. Italian Society of Gynecology and Obstetrics (President), Naples, Italy; 5 Department of Sciences for Health Promotion and Mother and Child Care “G. D’Alessandro”, University of Palermo, Palermo, Italy; 6 Department of Neuroscience–Rehabilitation–Ophthalmology-Genetics–Child and Maternal Health (DINOGMI), University of Genoa, Genoa, Italy; 7 Section of Pediatrics, Department of Translational Medical Science, “Federico II” University of Naples, Naples, Italy; 8 Unit of Emerging and Immunosuppressed Infectious Diseases, Department of Gastroenterology and Transplantation, Azienda Ospedaliero-Universitaria “Ospedali Riuniti”, Torrette Ancona, Rome, Italy; 9 Department of Pediatrics, Bambino Gesù Children’s Hospital, Rome, Italy; 10 AOGOI. Italian Association of Hospital Obstetricians-Gynecologists (President), Turin, Italy; 11 Department of Clinical Sciences and Community Health, University of Milan, Fondazione IRCCS Cà Granda Ospedale Maggiore Policlinico, Milan, Italy

**Keywords:** Breastfeeding, Vaccination, COVID-19

## Abstract

The availability of a COVID-19 vaccine has raised the issue of its compatibility with breastfeeding. Consequently, the Italian Society of Neonatology (SIN), the Italian Society of Pediatrics (SIP), the Italian Society of Perinatal Medicine (SIMP), the Italian Society of Obstetrics and Gynecology (SIGO), the Italian Association of Hospital Obstetricians-Gynecologists (AOGOI) and the Italian Society of Infectious and Tropical Diseases (SIMIT) have made an ad hoc consensus statement. Currently, knowledge regarding the administration of COVID-19 vaccine to the breastfeeding mother is limited. Nevertheless, as health benefits of breastfeeding are well demonstrated and since biological plausibility suggests that the health risk for the nursed infant is unlikely, Italian scientific societies conclude that COVID-19 vaccination is compatible with breastfeeding.

## Background: pandemic and vaccine

The COVID-19 pandemic was declared by the WHO on 11 March 2020 and Italy was the first country after China to be affected, counting, on 21 December 2020, 1.952.305 cases and 66.717 deaths [1].

During 2020, a huge scientific, organizational and economic effort has led to the availability of vaccines directed against SARS-CoV-2. Despite the acceleration of development, evaluation and approval times, these vaccines are effective, safe and of good quality to fight the pandemic. In Europe, four vaccines will come into use after approval by the European Medicines Agency (EMA): 2 mRNA vaccines containing genetic instructions to induce an immune response against coronavirus (BNT162b2; mRNA-1273) and 2 viral vector vaccines using harmless adenoviruses (ChAdOx1-SARS-CoV-2; Ad26.COV2.S) [2].

The public health strategies currently implemented at the international level imply the administration of the vaccine primarily to groups of population most at risk such as healthcare professionals and individuals aged 65 and over. The severity of the pandemic, however, led to shorten the development and evaluation period of COVID-19 vaccines and suggested an emergency regulatory authorization procedure. This acceleration has so far prevented from obtaining enough information on the safety of the COVID-19 among specific population groups such as children and adolescents less than 16 years, pregnant women and nursing women [3].

## Compatibility between breastfeeding and COVID-19 vaccination: indications from non-EU countries

UK. Initially, breastfeeding women, especially those belonging to the National Health Service (NHS), have been faced with the choice between continuing to breastfeed or postponing vaccination (“wait until you have finished breastfeeding before vaccinating yourself”) [4]. Instead, the UK Joint Committee on Vaccination and Immunization (JCVI) now advises that although “there is lack of safety data for these specific vaccinations in breastfeeding”, there is no known risk in giving these vaccines to breastfeeding women. Ultimately, “breastfeeding women should therefore be offered vaccination if they are otherwise eligible” [5, 6].

Canada. The Canadian Pfizer-BioNTech vaccine manufacturer sheet simply points out that it is not known whether the vaccine is excreted in human milk and that a risk to the newborn and infant cannot be excluded [7].

USA. The Food and Drugs Administration (FDA) [8, 9] and the Centers for Disease Control and Prevention (CDC) [10], rather than on the existence of a true contraindication, have emphasized the absence of scientific data: “no data are available to evaluate the effects of the Pfizer-BioNTech COVID-19 vaccine on the breast-fed infant or on milk production / excretion”. This position de facto leaves open the possibility of breastfeeding women being vaccinated. In fact:

- The US Society for Maternal-Fetal Medicine (SMFM) states that “the vaccine should be offered to women who are breastfeeding” and “are suitable in every other respect” [11];
- The American College of Obstetricians and Gynecologists (ACOG) recommends that COVID-19 vaccination also be offered to breastfeeding women, without the need to avoid vaccination or stop breastfeeding [12].
- The Academy of Breastfeeding Medicine (ABM) points out that “it is unlikely that the lipid component of the vaccine enters the bloodstream and reaches the breast tissue. If so, it is even less likely that an intact nanoparticle or mRNA will pass into the milk “ [13]. In the unlikely event that mRNA is found in milk, one would expect it to be digested by the baby without having any biological effect. In addition, the ABM notes, vaccination in breastfeeding would allow the passage from mother to infant of specific IgA antibodies against SARS-CoV-2 within 5–7 days [2].

EU. On 21 December 2020, the Committee for Medicinal Products for Use in Humans (CHMP) of the European Medicines Agency (EMA) issued a positive opinion on the safety and efficacy of the COVID-19 mRNA (Comirnaty) vaccine in the population aged 16 years and over. The EMA underlines that the vaccine mRNA is rapidly destroyed after the administration of the vaccine. The vaccine can be administered also to those with possible previous asymptomatic SARS-CoV-2 infection and to immunocompromised patients. Although data relating to the use of the vaccine during pregnancy are very limited, actually animal studies do not show dangerous effects. The decision whether or not to vaccinate during pregnancy should be made after consulting health professionals and assessing benefits and risks. Although currently no lactation study is ongoing, according to EMA no specific risk is expected for the breastfeeding mother and her/his nursed infant [14]. Finally, according to the manufacturer sheet, it is not known whether Comirnaty is excreted in human milk [15] and the breastfeeding woman is invited to refer to healthcare personnel. The Italian Medicines Agency has confirmed EMA’s indications [16].

## Compatibility between breastfeeding and COVID-19 vaccination: ad interim indications of the Italian scientific societies

On the basis of the above, a joint ad hoc Working Group of the Italian Society of Neonatology (SIN), the Italian Society of Pediatrics (SIP), the Italian Society of Perinatal Medicine (SIMP), the Italian Society of Obstetrics and Gynecology (SIGO), the Italian Association of Hospital Obstetricians-Gynecologists (AOGOI) and the Italian Society of Infectious and Tropical Diseases (SIMIT) have focused the issue of COVID-19 vaccination while breastfeeding. Between December 14 and 30, 2020 a facilitator gathered the appropriate knowledge, synthetized and balanced the varied opinions and suggestions from the other members of the Working Group. A general unanimous agreement on a preliminary draft was achieved by online communications from the entire panel. Eventually, the final version of the position statement has been approved by the Executive Committees of the adhering Scientific Societies.

Regarding the compatibility between breastfeeding and COVID-19 vaccination, the Italian Scientific Societies affirm that:

- Since breastfeeding must be promoted, protected and supported, due to the positive impact on maternal and child health, society and the environment [17–19], any contraindication to breastfeed should be based on precise medical reasons [20, 21].
- Breastfeeding women should not be systematically invited to stop breastfeeding in order to be vaccinated against COVID-19 [22]. In fact, the decision whether or not to administer the COVID-19 vaccine to the breastfeeding woman should be made after mutual agreement between her and the health professionals, taking into account specific health, social, familiar and work conditions.
- Biological plausibility suggests that in a breastfed baby the risk resulting from the mother’s COVID-19 vaccination is extremely low, while on the other hand, interruption of breastfeeding would lead to a certain loss of its well-documented benefits [23].
- Pregnant and lactating women should be included in future vaccination trials, particularly because pregnancy increases susceptibility to or severity of a disease [24] and because it represents a possible approach to protect the infant in the first months of life, following the transplacental transfer of anti-SARS-CoV-2 IgG antibodies [25].
- In conclusion, COVID-19 vaccination is currently considered compatible with breastfeeding.

## Abbreviations

COVID-19: Coronavirus disease 2019
mRNA: Messanger ribonucleic acid
